# Parasagittal dural space hypertrophy and amyloid-β deposition in Alzheimer’s disease

**DOI:** 10.1093/braincomms/fcad128

**Published:** 2023-04-18

**Authors:** Alexander K Song, Kilian Hett, Jarrod J Eisma, Colin D McKnight, Jason Elenberger, Adam J Stark, Hakmook Kang, Yan Yan, Ciaran M Considine, Manus J Donahue, Daniel O Claassen

**Affiliations:** Department of Neurology, Vanderbilt University Medical Center, Nashville, TN 37232, USA; Vanderbilt Brain Institute, Vanderbilt University, Nashville, TN 37232, USA; Department of Neurology, Vanderbilt University Medical Center, Nashville, TN 37232, USA; Department of Neurology, Vanderbilt University Medical Center, Nashville, TN 37232, USA; Department of Radiology and Radiological Sciences, Vanderbilt University Medical Center, Nashville, TN 37232, USA; Department of Neurology, Vanderbilt University Medical Center, Nashville, TN 37232, USA; Department of Neurology, Vanderbilt University Medical Center, Nashville, TN 37232, USA; Department of Biostatistics, Vanderbilt University Medical Center, Nashville, TN 32732, USA; Center for Quantitative Sciences, Vanderbilt University Medical Center, Nashville, TN 37232, USA; Department of Biostatistics, Vanderbilt University Medical Center, Nashville, TN 32732, USA; Department of Neurology, Vanderbilt University Medical Center, Nashville, TN 37232, USA; Department of Neurology, Vanderbilt University Medical Center, Nashville, TN 37232, USA; Vanderbilt Brain Institute, Vanderbilt University, Nashville, TN 37232, USA; Department of Neurology, Vanderbilt University Medical Center, Nashville, TN 37232, USA

**Keywords:** choroid plexus, parasagittal dural space, amyloid-β, cerebrospinal fluid, glymphatics

## Abstract

One of the pathological hallmarks of Alzheimer’s and related diseases is the increased accumulation of protein amyloid-β in the brain parenchyma. As such, recent studies have focused on characterizing protein and related clearance pathways involving perivascular flow of neurofluids, but human studies of these pathways are limited owing to limited methods for evaluating neurofluid circulation non-invasively *in vivo*. Here, we utilize non-invasive MRI methods to explore surrogate measures of CSF production, bulk flow and egress in the context of independent PET measures of amyloid-β accumulation in older adults. Participants (*N* = 23) were scanned at 3.0 T with 3D T_2_-weighted turbo spin echo, 2D perfusion-weighted pseudo-continuous arterial spin labelling and phase-contrast angiography to quantify parasagittal dural space volume, choroid plexus perfusion and net CSF flow through the aqueduct of Sylvius, respectively. All participants also underwent dynamic PET imaging with amyloid-β tracer ^11^C-Pittsburgh Compound B to quantify global cerebral amyloid-β accumulation. Spearman’s correlation analyses revealed a significant relationship between global amyloid-β accumulation and parasagittal dural space volume (rho = 0.529, *P* = 0.010), specifically in the frontal (rho = 0.527, *P* = 0.010) and parietal (rho = 0.616, *P* = 0.002) subsegments. No relationships were observed between amyloid-β and choroid plexus perfusion nor net CSF flow. Findings suggest that parasagittal dural space hypertrophy, and its possible role in CSF-mediated clearance, may be closely related to global amyloid-β accumulation. These findings are discussed in the context of our growing understanding of the physiological mechanisms of amyloid-β aggregation and clearance via neurofluids.

## Teaser

Song *et al*. report that parasagittal dural (PSD) space hypertrophy, specifically in frontal and parietal subsegments, is associated with increasing amyloid-β accumulation in Alzheimer’s disease patients. These results suggest that PSD space may be related to cerebrospinal fluid egress and amyloid-β waste clearance.

## Graphical Abstract

**Graphical abstract:**
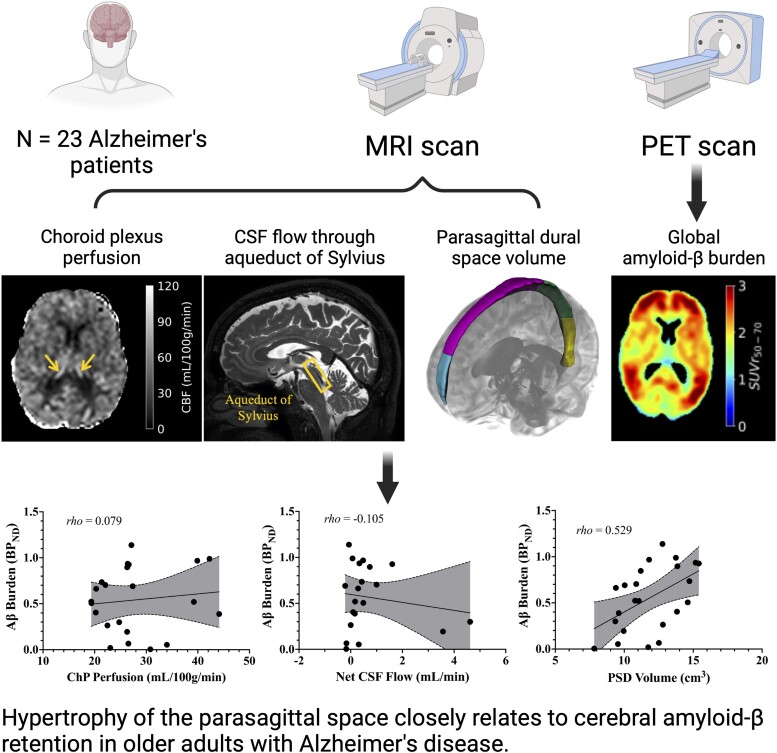

## Introduction

Alzheimer’s disease is pathologically characterized by increased cerebral accumulation of amyloid-β (Aβ) and tau aggregates. Aβ accumulation can be identified using PET radioligands such as ^18^F-florbetapir and ^11^C-Pittsburgh Compound B (PIB) and evidence of increased Aβ has been reported in the cingulate, frontal, parietal and temporal cortices; however, the underlying aetiology of Aβ accumulation is incompletely characterized.^1-6^ Aβ clearance dysfunction from the CNS likely contributes to Aβ retention and factors underlying Aβ clearance include, but are not limited to, enzymatic degradation,^7^ transport across the blood–brain and CSF–blood barriers,^8^ interstitial fluid (ISF) bulk flow^9^ and CSF egress.^10,11^

More specifically, CSF production primarily occurs in the choroid plexus (ChP) complexes, an extension of the ependymal epithelium located in the brain’s ventricles.^12^ The majority of CSF production occurs in the lateral and third ventricles, after which this CSF flows caudally through the aqueduct of Sylvius to the fourth ventricle and subsequently to the subarachnoid space (SAS) through the foramen of Magendie and Luschka, after which a portion of this fluid transits to the dural venous sinuses to be resorbed into the blood circulation via arachnoid granulations. There is increasing evidence that CNS waste product clearance may also occur along perivascular and interstitial spaces.^11^ In this explanation, CSF flows from the SAS to the periarterial space where it then transits to interstitial space, as governed by periarterial aquaporin 4 channels (AQP4).^11,13,14^ It has been proposed that fluid motion in the brain parenchymal interstitial space moves fluid into the perivenous space via AQP4 channels.^15^ Very recently, CSF egress evaluated using intrathecally administered contrast agents has also been proposed to occur along the parasagittal dural (PSD) space which surrounds the sagittal sinus.^16-18^ While features of these pathways remain to be elucidated, proximal and distal features of the neurofluid circulation, comprising ChP activity, CSF flow through the aqueduct and PSD anatomy likely have relevance to the clearance of neurofluids from the CNS.

**Figure 1:**
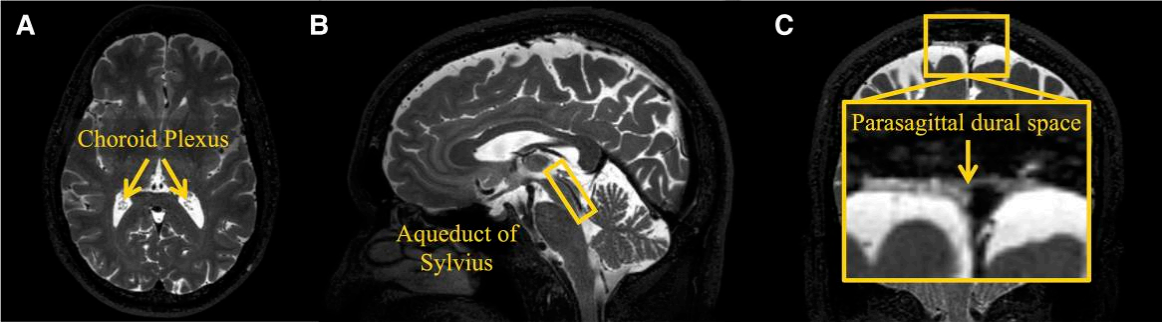
**Components of neurofluid circulation.** Orthogonal view of a 3D T_2_-weighted VISTA image highlighting the different components of neurofluid circulation including the **(A)** ChP in the axial slice, **(B)** aqueduct of Sylvius in the sagittal slice and **(C)** PSD space in the coronal slice.

We have recently reported on novel MRI methods to quantify these measurements in humans *in vivo* and non-invasively.^19,20^ In previous work, we observed age-related hypertrophy of both the ChP and PSD. We also observed decreases in ChP perfusion and caudal CSF flow through the aqueduct of Sylvius with increasing age. Here, we apply measures of ChP activity, net CSF flow, PSD volume and Aβ plaque accumulation in sequence in patients with Alzheimer’s disease to test the hypotheses that patients with higher levels of Aβ burden have: (i) reduced ChP perfusion (Fig. 1A), (ii) reduced net CSF flow through the aqueduct of Sylvius (Fig. 1B) and (iii) hypertrophy of the PSD space (Fig. 1C). Findings are discussed in the context of the growing field of neurofluid dysfunction and proteinopathy.

## Materials and methods

### Participants

All participants provided written, informed consent in accordance with the institutional review board at Vanderbilt University Medical Center and the Declaration of Helsinki and its amendments. Participants were recruited from the clinical services of the Vanderbilt University Medical Center Behavioral and Cognitive Neurology clinics.

The inclusion criteria for the study included participants between the ages of 55 and 80 years (inclusive) with clinic-pathological diagnosis of either amnestic mild cognitive impairment (aMCI) due to Alzheimer’s disease-intermediate likelihood or probable Alzheimer’s dementia-intermediate likelihood, per criteria based on recommendations from the National Institute on Aging and Alzheimer’s Association workgroups diagnostic guidelines.^21,22^ The aMCI criteria integrated combined Jak/Bondi (2 + memory performances <1.0 SD) and Petersen/Winblad (1 memory measure <1.5 SD) diagnostic frameworks.^23^ Clinical criteria were based on consensus determination following examination by a board-certified neurologist (D.O.C.) and board-certified neuropsychologist (C.M.C.) which includes a Clinical Dementia Rating (CDR) as well as a multi-domain cognitive exam [Montreal Cognitive Assessment; Repeatable Battery for the Assessment of Neuropsychological Status (RBANS)].^24-26^ Biomarker probability criteria were based on Aβ PET read by a board-certified neuroradiologist (C.D.M.). Participants were excluded from the study if they had any contraindications to either 3 T MRI or PET as defined by the institutional standard screening criteria including pregnancy and ferrous metal implants. Participants were also excluded if radiological findings consistent with an independent neurological and/or neurovascular condition (including but not limited to overt stroke, multiple sclerosis or brain tumour) were observed upon MRI review.

### Cognitive evaluation

Immediately prior to imaging, participants underwent a neuropsychological examination consisting of the Montreal Cognitive Assessment and RBANS to characterize cognitive status. The Montreal Cognitive Assessment is a cognitive screen that incorporates items with appropriate range of difficulty to provide sensitivity to MCI and facilitate differentiation of dementia stage deficits, while also assessing a breadth of cognitive domains beyond memory, which allows for improved sensitivity to atypical/non-amnestic cognitive syndromes.^27^ The RBANS is a clinical neuropsychological test battery designed specifically for the diagnosis and longitudinal monitoring of cognitive status.^26^ The measure was designed to optimize sensitivity for the detection of prodromal manifestation of dementia while remaining brief enough in administration length to lend itself to diverse clinical and research assessment settings. The RBANS generates age-normed scaled or percentile scores for all subtests, collapsed into index scores for five cognitive domains, along with a global cognitive functioning total index score. Clinical research has established strong associations between RBANS and performance on longer-length neuropsychological batteries, the six domains of the CDR, clinical–functional diagnosis of MCI and dementia syndromes, biomarkers in Alzheimer’s disease, as well as response to intervention in clinical trials, making the measure a gold-standard of cognitive-behavioural phenotyping in MCI/dementia research.^28^

### Magnetic resonance imaging

Participants were scheduled for same-day MRI and PET scans between 8:00 am and 11:00 am. All participants were scanned at 3T (Philips Healthcare, Best, The Netherlands) using a body coil radiofrequency transmission and phased array 32-channel reception with the placement of four MRI-compatible ECG leads for cardiac phase correction as described below. The scanning protocol consisted of a 3D T_1_-weighted magnetization-prepared-rapid-gradient-echo (TR = 8.1 ms, TE = 3.7 ms, spatial resolution = 1.0 × 1.0 × 1.0 mm^3^) for anatomical reference, 3D T_2_-weighted volume isotropic-turbo-spin-echo-acquisition (TR = 2500 ms, TE = 331 ms, spatial resolution = 0.78 × 0.78 × 0.78 mm^3^) for PSD volumetric measurement, and 2D T_2_-weighted fluid-attenuated inversion recovery (FLAIR; TR = 11 000 ms, TE = 120 ms, TI = 2800 ms, spatial resolution = 0.57 × 0.57 × 4.0 mm^3^) turbo-spin-echo for ChP segmentation. A 2D dual-pulse background-suppressed pseudo-continuous arterial spin labelling (labelling duration = 1800 ms, PLD = 2000 ms, TR = 4550 ms, TE = 11 ms, spatial resolution = 3.0 × 3.0 × 7.0 mm^3^) sequence was applied to quantify ChP and cortical perfusion. A 2D readout was used to reduce artefacts from signal smearing in the phase encode direction, common in 3D GRASE readouts, to ensure accurate localized blood flow quantification in the ChP. For this scan, an equilibrium magnetization (M_0_) scan was sequentially acquired with identical geometry and scanner gain, but with pseudo-continuous arterial spin labelling preparation removed and TR = 15 000 ms. A phase-contrast angiography acquisition (2D gradient echo; TR = 12.0 ms, TE = 7.8 ms, spatial resolution = 0.59 × 0.59 × 4.0 mm^3^, velocity encoding = 12 cm/s) was adapted to quantify net CSF flow draining from the lateral and third ventricles at the level of the aqueduct of Sylvius.^20^

### Positron emission tomography

All PET scans were acquired using a Philips Vereos Digital PET/CT scanner (Philips Healthcare, Best, The Netherlands) with 3D emission acquisition. First, a 5 min transmission scan was also performed to enable attenuation correction. Next, following an intravenous bolus injection of ^11^C-PIB, a 70 min dynamic PET scan was acquired (frames: 4 × 15 s, 8 × 30 s, 9 × 60 s, 2 × 180 s and 10 × 300 s). Data from the PET scans were reconstructed with a 3D-FRP (direct Fourier method with Fourier reprojection) algorithm for dynamic and a 3D-ordered subset expectation maximum algorithm for static analyses, achieving an isotropic 2.0 mm reconstructed image spatial resolution.

### MRI analysis

The goal of the MRI analysis was to provide quantitative measurements of ChP perfusion (ml/100 g/min) from arterial spin labelling MRI, net CSF flow (ml/min) from phase-contrast MRI and PSD volume (cm^3^) from 3D T_2_-weighted MRI.

#### ChP perfusion

**Figure 2:**
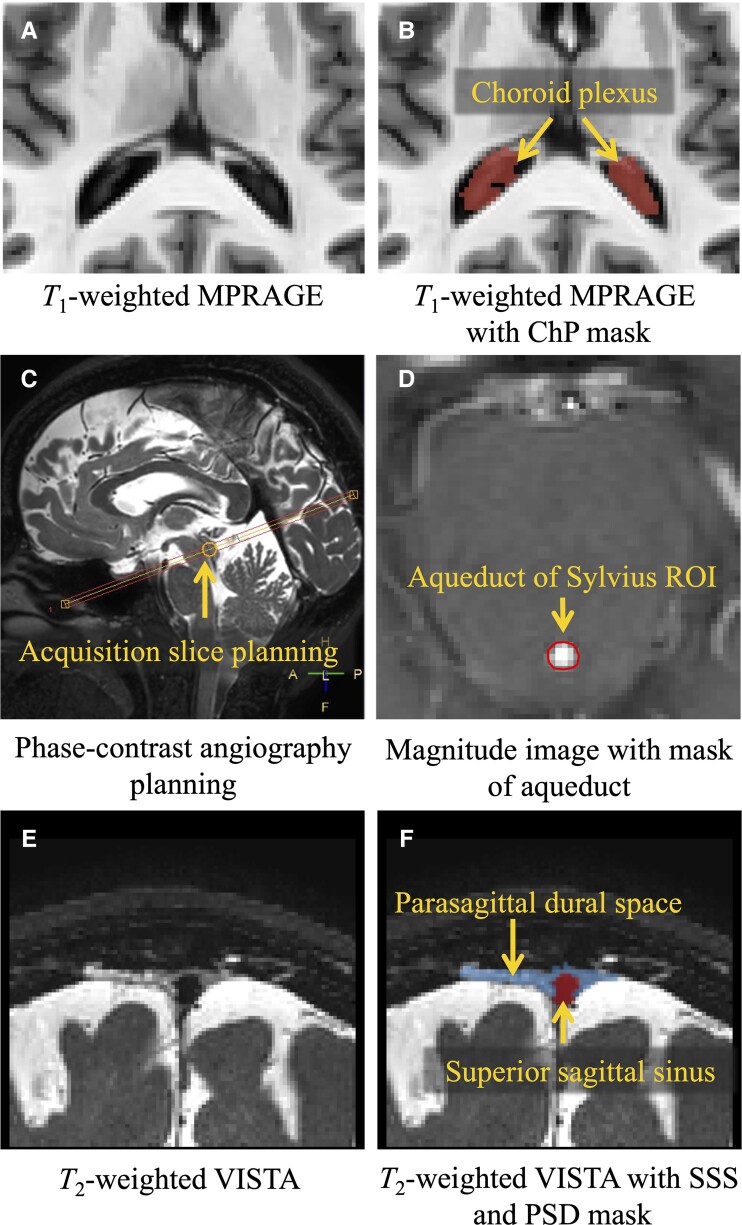
**Overview of MR images and processing results.** (**A** and **B**) Example axial view of a T_1_-weighted MPRAGE image and the resulting ChP (red) mask generated from the F-CNN for automatic segmentation of the ChP. (**C** and **D**) Example image of the phase-contrast acquisition planning on a sagittal view of T_2_-weighted VISTA image and an axial view of the magnitude image from acquisition to delineate the cerebral aqueduct (red) for use in the calculation of net CSF flow. (**E** and **F**) Coronal view of a T_2_-weighted VISTA image and the output mask (blue, PSD space; red, superior sagittal sinus) from the semi-supervised machine learning method using an F-CNN for automatic segmentation of the parasagittal space.

ChP perfusion was calculated using a previously published deep-learning segmentation pipeline.^20^ Briefly, participants’ T_1_-weighted and T_2_-weighted FLAIR images were transformed to 1 mm3 isotropic MNI152 space using the Advanced Normalization Tools non-linear registration tool.^29^ The ChP was then automatically segmented using a fully convolutional neural network (F-CNN) based on a 3D U-Net architecture adapted from Zhao *et al*.^30^ The neural network was trained on a training data set of 30 manual ChP segmentations that were visually inspected by a board-certified neuroradiologist (C.D.M.) for accuracy. The generated output masks were then back-transformed to native T_1_-weighted space for ChP perfusion quantification (Fig. 2A and B). Perfusion maps were generated from pre-processing of source pseudo-continuous arterial spin labelling control and label images. These pre-processing steps included surround subtraction, slice delay correction, equilibrium magnetization normalization, averaging and simplified general kinetic model fitting.^31^ The processed perfusion maps were then aligned to native T_1_-weighted space, using rigid transformation (six degrees of freedom) estimated with a skull-stripped T_1_-weighted MPRAGE image, to calculate mean perfusion (ml/100 g/min) within the generated ChP masks.

#### CSF flow in the cerebral aqueduct

Volumetric flow parameters of CSF through the aqueduct of Sylvius were derived from acquired phase-contrast data that were retrospectively cardiac phase-corrected (12 cardiac phases) from the ECG traces during acquisition. The sequence was planned such that the acquisition slice was orthogonal to the aqueduct of Sylvius in the sagittal view (Fig. 2C). A mask of the aqueduct of Sylvius was manually delineated from the magnitude image (Fig. 2D) and subsequently applied to the phase image in the phase-contrast acquisition to obtain the acquired phase of moving CSF in the region throughout the cardiac cycle. The net flow (ml/min) was calculated by integrating the CSF flow curve across time and multiplying by the participant’s mean heart rate to account for variations in the duration of cardiac cycle across participants. It should be noted that as the aqueduct at the level of the tectum is surrounded by structures without movement, the flux calculation is largely insensitive to the size of the region drawn so long as the size is at or larger than the cross-sectional area of the aqueduct (since the flux of the surrounding non-flowing tissue is null). A phase aliasing correction was also applied to correct for phase values from the participants with flow velocities greater than the velocity encoding value of 12 cm/s. The net flow parameter represents the net volume of CSF that flows out of the third ventricle and through the aqueduct of Sylvius per minute.^20^

#### PSD space volume

PSD segmentation masks were generated using participant 3D T_2_-weighted scans (Fig. 2E and F).^19^ An N4 inhomogeneity correction was applied to participant 3D T_2_-weighted images to reduce bias field inhomogeneity, and the images were then transformed to MNI ICBM-152 T_2_W template using Advanced Normalization Tools.^32,33^ A semi-supervised machine learning method was used to automatically segment the PSD based on a combination of an F-CNN. The method relies on a stacked U-Net architecture where the first layer estimates a binary mask of the peri-sinus space and the second layer delineates a label map of PSD and superior sinus lumen based on T_2_-weighted signal intensities. The neural network was trained on a data set consisting of 20 manual segmentations of the parasagittal space on T_2_-weighted images by a board-certified neuroradiologist (C.D.M.). The PSD was further subdivided into four subregions: the prefrontal, frontal, parietal and occipital PSD. The prefrontal PSD and the frontal PSD are distinguished by a plane crossing through the pituitary gland and the rostrum of the corpus callosum such that the prefrontal PSD lies ventral to the plane and the frontal PSD lies dorsal to the plane. The parietal region was delineated from the frontal PSD using the central sulcus and extends to the parietal–occipital fissure; finally, the occipital PSD was delineated from the parietal–occipital fissure to the most posterior portion of the PSD. The resulting masks were then transformed back to native T_2_-weighted space using the inverse transform. In this work, we investigated total and regional PSD volumes all expressed in cubic centimetres (cm^3^).

### PET analysis

Participant T_1_-weighted MPRAGE images were used for whole brain segmentation using the AssemblyNet segmentation framework.^34^ A global region of interest mask of cortical and subcortical regions susceptible to Aβ accumulation was created with the segmentation output similar to the global mask used in Klunk *et al*.^35^ A list of the brain regions included in this mask is outlined in Supplementary Table 1. The resulting global mask was used for subsequent quantitative analyses of Aβ burden.

Parametric standardized uptake value (SUV) maps were calculated from the last 20 min of the scan (50–70 min post-injection; frames: 4 × 300 s). The PMOD software package (version 4.2, PMOD Technologies LLC, Zürich, Switzerland) was used to evaluate the SUV on a voxel-wise basis. Participant SUV data were rigidly registered to native T_1_-weighted space for partial volume correction and Aβ quantification using FSL’s FLIRT with six degrees of freedom and mutual information cost function (FSL v6.0, FMRIB, Oxford, UK). Region-based voxel-wise (RBV) partial volume correction was then applied to the registered SUV data.^36^ Global mean values for SUV were calculated using the segmented global mask and a 10% trimmed mean value to improve robustness.^36^ The global mean SUV was then normalized to cerebellar grey matter (GM) SUV (Global SUV/Cerebellar GM SUV) to account for non-specific tracer binding and generate a SUV ratio (SUVr). Several studies have demonstrated that the use of the cerebellar GM as a reference region in quantifying Aβ burden with ^11^C-PIB produced the largest effect sizes in differentiating Aβ-positive and -negative scans.^37^

Parametric images of binding potential were generated using a two-step simplified reference tissue model (SRTM2).^38^ The first step uses the simplified reference tissue model to fit three parameters: non-displaceable binding potential (BP_ND_), relative tracer delivery (R1) and reference region efflux constant ($k_{2}^{\prime }$). The $k_{2}^{\prime }$ parameter is then fixed to the median $k_{2}^{\prime }$ value of all voxels in the brain with a BP_ND_ value >0.1.^39^ A final BP_ND_ map is generated using the fixed $k_{2}^{\prime }$ parameter. Again, the cerebellar GM was used as a reference region for parametric binding potential estimation. A mean reference image of motion-corrected serial PET frames was created and used to rigidly register the BP_ND_ image to native participant T_1_-weighted MR space. RBV partial volume correction was then applied to the registered BP_ND_ image. Global mean values for BP_ND_ were calculated using the segmented global mask and a 10% trimmed mean value.

### Statistical analyses

Statistical analyses were performed using the R software package (version 4.2.1, R Foundation for Statistical Computing, Vienna, Austria). Anderson–Darling Goodness of Fit tests were applied to all quantitative imaging data to test if the data are normally distributed. We performed the following separate analyses with Spearman’s rank-order correlation tests:

1. To understand the similarity of BP_ND_ quantified by SRTM2 in subsequent analyses, we assessed the agreement of global Aβ burden with static (SUVr_50–70_) and dynamic (BP_ND_ + 1) models. BP_ND_ + 1, equivalent to the distribution volume ratio, was used in this specific hypothesis for direct comparison with SUVr_50–70_ as previously published.^40^
2. To test the primary hypotheses, we assessed the relationship between ^11^C-PIB BP_ND_ and the imaging biomarker of interest (ChP perfusion, net CSF flow through the aqueduct of Sylvius and total PSD volume). Significance criteria for all primary hypotheses were set at *P* < 0.05.
3. In an exploratory analysis of the relationship between global Aβ burden and specific subregions of the PSD, we assessed the correlation between global ^11^C-PIB BP_ND_ and subsegmental PSD volumes. Multiple comparison corrections were applied for *post hoc* analyses with a false discovery rate (FDR)-calculated *P* < 0.05.^41^
4. To test whether PSD hypertrophy was a product of general brain atrophy, we assessed the correlation between cumulative grey matter and white matter (GM + WM) tissue volume and total PSD volume. We also assessed this relationship with volumes adjusted for total intracranial cavity volume to account for variations in intracranial size.

Supplementary analyses of the above hypotheses using partial correlations were also used to test all hypotheses while covarying for age and sex; results are provided in Supplementary Table 2.

## Results

### Demographics and quantitative imaging measurements

**Figure 3:**
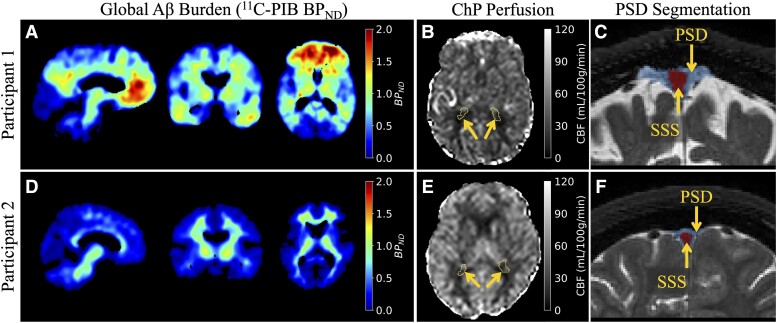
**Measures of Aβ and neurofluid circulation in participants with higher and lower Aβ burden.** Participant 1 is a 73-year-old male with the highest Aβ levels as determined by ^11^C-PIB binding (**A**; global BP_ND_ = 0.935). Corresponding axial slice of ChP (yellow arrows) cerebral blood flow (**B**; ChP perfusion = 26.41 ml/100 g/min) and coronal slice of PSD (blue) and superior sagittal sinus (SSS; red) segmentation (**C**; volume = 15.17 cm^3^) are displayed. Participant 2 is a 56-year-old female with the lowest Aβ levels as determined by ^11^C-PIB binding (**D**; global BP_ND_ = 0.005). Corresponding axial slice of the ChP (yellow arrows) cerebral blood flow (**E**; ChP perfusion = 30.79 ml/100 g/min) and coronal slice of PSD (blue) and SSS (red) segmentation (**F**; volume = 7.81 cm^3^) are displayed.

A total of 23 participants ranged in age from 56 to 78 years (mean = 70.0 ± 6.0 years) and were comprised of 10 males and 13 females. All participants met neurological and radiological inclusion criteria as defined above. The mean Montreal Cognitive Assessment and RBANS scores for all participants were 18.91 ± 4.80 and 72.57 ± 18.30, respectively. A summary of the demographics and quantitative imaging measurements is outlined in Table 1. All quantitative imaging measurements were normally distributed, except for ChP perfusion (adjusted A2 = 0.952, *P* = 0.016) and net CSF flow (adjusted A2 = 3.200, *P* < 0.001). Figure 3 shows representative voxel-wise BP_ND_ maps obtained from SRTM2 for a participant with higher and lower Aβ levels alongside corresponding ChP perfusion maps and PSD segmentation masks.

**Table 1:** Demographic and imaging characteristics

Variable	Mean ± SD	Range
Age (years)	70.0 ± 6.1	56–78
MoCA score (total)	18.91 ± 4.80	5–26
RBANS score (total)	72.57 ± 18.30	45–109
Global Aβ burden (SUVr)^a^	2.06 ± 0.52	1.04–2.80
Global Aβ burden (BP_ND_)	0.553 ± 0.349	0.005–1.14
ChP volume (cm^3^)^a^	4.297 ± 2.017	2.12–11.11
ChP perfusion (ml/100 g/min)^a^	27.72 ± 2.16	19.35–44.14
Net CSF flow (ml/min)^b,c^	0.686 ± 1.254	−0.216–4.617
Total PSD volume (cm^3^)	11.85 ± 2.16	7.81–15.41
Prefrontal PSD (cm^3^)	0.41 ± 0.24	0.00–1.02
Frontal PSD (cm^3^)	5.45 ± 1.11	3.65–7.37
Parietal PSD (cm^3^)	3.34 ± 0.77	2.03–4.88
Occipital PSD (cm^3^)	2.66 ± 0.57	1.36–3.82

*N* = 23 unless otherwise noted.

BP_ND_, non-displaceable binding potential; ChP, choroid plexus; MoCA, Montreal Cognitive Assessment; PSD, parasagittal dural; RBANS, Repeatable Battery for the Assessment of Neuropsychological Status; SUVr, standardized uptake value ratio.

a *n* = 22.

b *n* = 20.

c The directionality of negative CSF flow is defined as anterograde (caudal-to-cranial) movement of CSF through the aqueduct of Sylvius.

### PET quantification

**Figure 4:**
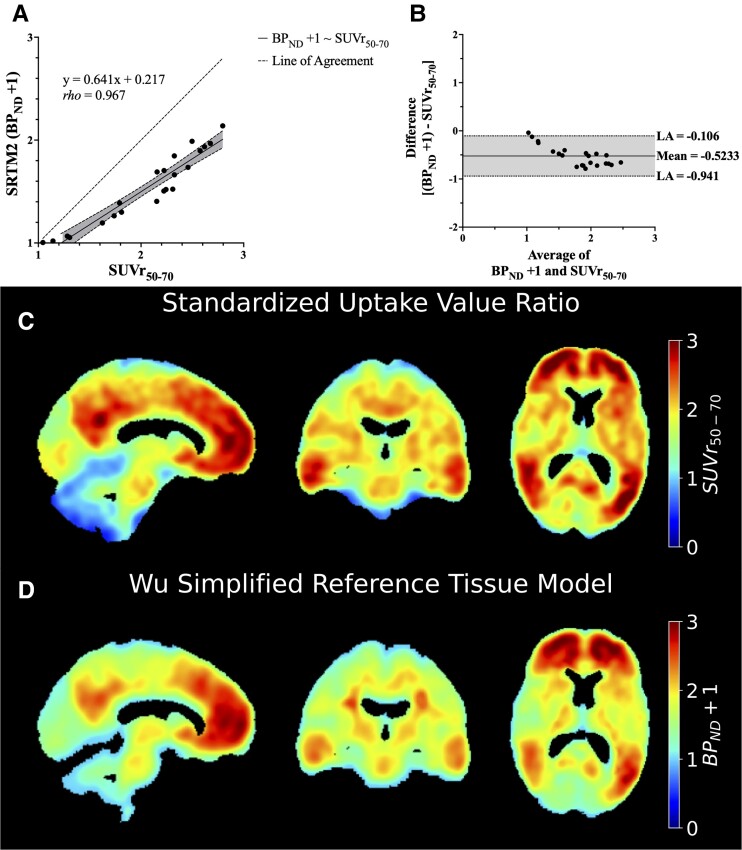
**Comparison of static and dynamic analyses of ^11^C-PIB.** (**A**) The scatterplot of SUVr_50–70_ and corresponding SRTM2 (BP_ND_ + 1) values derived from ^11^C-PIB PET imaging in 22 participants is displayed. Spearman’s rank-order correlation analysis demonstrates a significantly positive correlation (rho = 0.967, *P* < 0.001) between the two measures of Aβ accumulation. (**B**) The Bland–Altman plot of the average against the difference of SUVr_50–70_ and SRTM2 values reveals that SUVr_50–70_ values are consistently higher than corresponding SRTM2 BP_ND_ + 1 values. Orthogonal views of parametric ^11^C-PIB maps in an example participant are displayed for the (**C**) static SUVr_50–70_ and (**D**) dynamic SRTM2 analysis methods.

Twenty-two out of 23 participants were included in the comparison of static SUVr_50–70_ and dynamic SRTM2 BP_ND_ values due to a participant leaving the PET scanner early. Global levels of Aβ burden as measured by SUVr_50–70_ and BP_ND_ + 1 were closely associated and met significance criteria (rho = 0.967, *P* < 0.001). A scatterplot of SUVr_50–70_ and BP_ND_ + 1 and the Bland–Altman plot are displayed in Fig. 4 alongside corresponding example images. Thus, all subsequent analyses used BP_ND_ as the representative measure for global Aβ burden.

### Global Aβ burden and CSF production, flow and PSD volume

**Figure 5:**
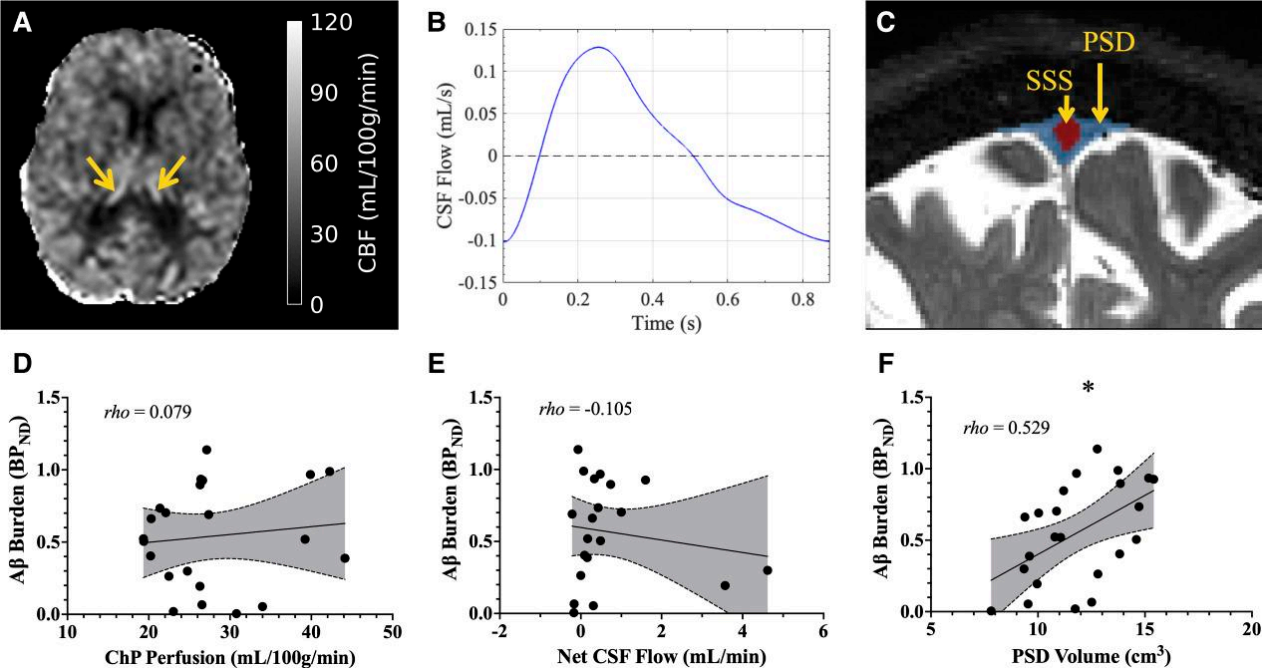
**Scatterplots of global Aβ burden and measures of neurofluid circulation.** (**A**) Axial view of ChP is shown in ChP is indicated to with yellow arrows. (**B**) Net CSF flow is calculated as the integral of the CSF flow time course shown. (**C**) The segmentation of the PSD is outlined in blue and the superior sagittal sinus in red. Corresponding scatterplots for global Aβ burden against **(D**) ChP perfusion, (**E**) net CSF flow and (**F**) PSD volume are also displayed. Spearman’s rank-order correlation analyses reveal no significant relationship between global Aβ burden and ChP perfusion (**D**; rho = 0.079, *P* = 0.748) or net CSF flow (**E**; rho = −0.105, *P* = 0.668). The relationship between global Aβ burden and PSD volume was found to be significant (**F**; rho = 0.529, *P* < 0.001).

Analysis of ChP perfusion and global Aβ burden included 22 out of 23 participants due to the pseudo-continuous arterial spin labelling sequence not being acquired in one participant. Examples of ChP perfusion, CSF flow through the aqueduct and PSD volume are displayed in Fig. 5A–C. ChP perfusion was not closely associated with global BP_ND_ values of ^11^C-PIB and did not meet significance criteria (rho = 0.079, *P* = 0.748, *n* = 22; Fig. 5D). Analysis of net CSF flow and global Aβ burden included 21 out of 23 participants due to poor ECG trace during image acquisition in two participants. The relationship between net CSF flow at the aqueduct of Sylvius and global BP_ND_ of ^11^C-PIB also did not meet significance criteria (rho = −0.105, *P* = 0.668, *n* = 20; Fig. 5E). Analysis of total PSD space volume and global Aβ burden included all 23 participants. Total PSD volume was positively associated with global BP_ND_ values and met the significance criteria (rho = 0.529, *P* = 0.010, *n* = 23; Fig. 5F).

### Global cortical Aβ burden and PSD subsegments

**Figure 6:**
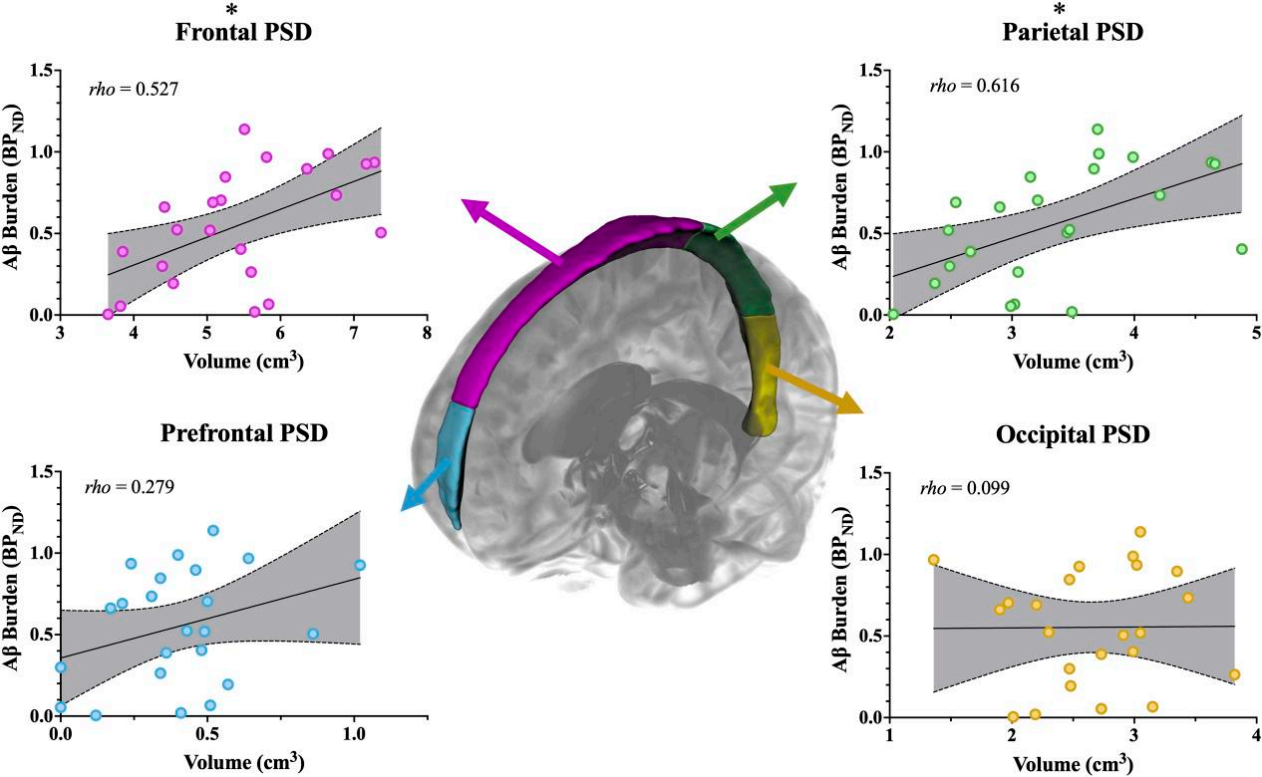
**Relationships between global Aβ burden and subsegmental PSD space volume.** A 3D rendering of the PSD space and its subregions is depicted with associated scatterplots for the prefrontal (blue), frontal (magenta), parietal (green) and occipital (yellow) subregions of the PSD. Each scatterplot includes a best-fit line and 95% confidence intervals (shaded grey). Spearman’s rank-order correlation analyses revealed that increases in PSD volume of the frontal and parietal subregions of the PSD were significantly associated with increasing ^11^C-PIB BP_ND_ (rho = 0.527, *P* = 0.010; rho = 0.616, *P* = 0.002, respectively). These results survived multiple comparison correction.

A schematic of the PSD subregions and associated scatterplots for each subregion is depicted in Fig. 6. Both the frontal and parietal PSD volumes were positively associated with global BP_ND_ of ^11^C-PIB, met significant criteria (frontal: rho = 0.527, *P* = 0.010; parietal: rho = 0.616, *P* = 0.002), and survived multiple comparison corrections of *p*_FDR_ < 0.05. The prefrontal and occipital PSD volumes were not closely associated with global BP_ND_ (prefrontal: rho = 0.279, *P* = 0.197; occipital: rho = 0.099, *P* = 0.653). Overall, increases in PSD volume of specifically the frontal and parietal subregions were significantly associated with increases in global BP_ND_ of ^11^C-PIB.

### PSD hypertrophy and general brain atrophy

Correlation analyses revealed that total GM + WM tissue volume was not significantly associated with total PSD volume (rho = −0.17; *P* = 0.441). Additionally, this relationship remained statistically insignificant after corrections for total intracranial volume (rho = 0.344; *P* = 0.114).

## Discussion

We performed sequential MRI and PET in patients along the clinicopathological spectrum of Alzheimer’s disease to provide preliminary information on relationships between quantitative measures Aβ protein accumulation and MR imaging markers related to CSF production, flow and egress. We used dynamic ^11^C-PIB imaging to quantify Aβ burden; pseudo-continuous arterial spin labelling imaging and an F-CNN segmentation method of T_1_-weighted MPRAGE images to quantify ChP perfusion; a novel application of phase-contrast angiography imaging to quantify net CSF flow through the aqueduct of Sylvius; and an F-CNN segmentation method to automatically delineate the PSD on T_2_-weighted VISTA images and quantify PSD volume. Our main finding was that hypertrophy of the total PSD space was significantly associated with elevated global Aβ burden; specifically, hypertrophy of the frontal and parietal subsegments of the PSD contributed to the relationship with Aβ levels. In contrast, ChP perfusion and net CSF flow through the aqueduct of Sylvius were not associated with Aβ accumulation. The present findings suggest that the PSD space, which has previously been implicated in CSF egress, may have a significant role with respect to the accumulation of Aβ. These results may be used to help inform the direction of future studies of PSD hypertrophy, neurofluid circulation and proteinopathy.

One emerging concept proposes that disruptions to perivascular and interstitial neurofluid flow (e.g. glymphatic flow) and waste clearance activity may predispose individuals to excessive protein aggregation and contribute to the pathophysiology of Alzheimer’s disease. Consistent with this possibility, Li *et al*.^42^ have shown reduced ventricular CSF clearance was associated with increased Aβ concentrations in Alzheimer’s disease, suggesting that failed CSF clearance may be a key feature of Alzheimer’s disease that relates to the protein aggregations seen in the pathology of the disease.^43^ Furthermore, a stable isotope-labelled kinetic study reported increased Aβ_42_, but reduced Aβ_38/40_, turnover in amyloid-positive compared with amyloid-negative participants.^44^ Under the assumption that blood–brain barrier transport and proteolytic mechanisms of Aβ_38/40_ and Aβ_42_ are similarly downregulated, this finding suggests a greater emphasis is placed on CSF clearance mechanisms to remove Aβ.

Previous studies have demonstrated a strong ageing effect with PSD volume, suggesting a potential compensatory mechanism or alternative evidence of decompensatory tissue dysfunction in response to worsening CSF or ISF drainage with age.^19,45^ Here, for the first time, we report a significant relationship between PSD volume and global Aβ protein aggregation, further suggesting that increased Aβ protein aggregation may relate to dysfunctional CSF clearance. Interestingly, we observed the strongest relationship between global Aβ accumulation and PSD volume in the frontal and parietal subsegments. These regions correlate with commonly observed Aβ aggregation topography in the frontal cortex, anterior and posterior cingulate, and precuneus. In contrast, we did not observe similar relationships in the prefrontal and occipital PSD subsegments. Additionally, PSD volume was not associated with whole brain tissue volume, even when correcting for total intracranial cavity volume, suggesting that hypertrophy of the PSD occurs independently of general brain atrophy. Supporting this, ultrastructural electron microscopy of human dura samples revealed that dural channels were more widely distributed in elderly patients than in younger patients, who exhibited densely concentrated channels around the superior sagittal sinus.^18^ These dural channels were found to lack expression of lymphatic and vascular markers, suggesting a potential reservoir-like role for CSF drainage.

Indeed, in human studies with intrathecal injection of gadobutrol, an MRI contrast agent serving as a CSF tracer, and high-resolution MRI demonstrated an efflux of gadobutrol into the PSD with peak enrichment at 24 h post-injection in humans.^16^ In comparison, minimal efflux of tracer was found in the cribriform plate, another hypothesized CSF clearance pathway. Animal studies have also previously shown that complete aplasia of dural lymphatic vessels results in attenuated macromolecule clearance and abolished transport of CSF from the SAS to deep cervical lymph nodes.^46^ Together with the present findings, it is evident that the PSD plays an important, yet unclear role in CSF clearance and egress. A possible explanation for PSD hypertrophy may be a compensatory mechanism in response to increasing Aβ for the PSD to promote greater clearance by increasing communication with the superior sagittal sinus and/or providing increased collateral communication with alternate routes of CSF clearance such as along the cranial nerves. This may lead to channels within the frontal and parietal subsegments of the PSD space to become dilated and/or more diffusely distributed to promote greater CSF efflux. Alternatively, neuroinflammation has emerged as a possible link between Aβ and tau proteinopathies;^47^ and these inflammatory markers may trigger a hypertrophic response in the PSD. While the mechanisms with which these dural channels along the superior sagittal sinus are involved in CSF egress and waste clearance are not yet fully understood, it is evident that the PSD plays an important role in CSF egress, and further characterization of these channels is necessary to elucidate the potential implications of Alzheimer’s disease and neurodegenerative proteinopathies.

We have previously shown age-related reductions in ChP perfusion and net CSF flow through the aqueduct of Sylvius in healthy adults, and further demonstrated a positive relationship between these two measures.^20^ These findings provided support that CSF production from the ChP can be detected downstream in the CSF flow pathway and allowed us to establish surrogate markers of choroidal CSF production. In the present study, we did not observe relationships with global Aβ accumulation and either of the two measures, which may indicate that choroidal CSF production is not involved in the clearance of Aβ. Previous studies have suggested the involvement of ChP dysfunction in impaired Aβ clearance.^48,49^ The ChP is selectively permeable to Aβ in the CSF-facing membrane of the blood–CSF, barrier, thus suggesting an important role for the ChP in Aβ clearance other than CSF production. In support of this, a triple transgenic mouse model of Alzheimer’s disease demonstrated increased deposition of specifically isoform Aβ_42_ in the ChP epithelial cytoplasm, reflecting insufficient clearance transport from CSF to blood. It is possible that age-related downregulation of choroidal CSF production places a greater burden on waste transport via CSF clearance pathways, such as bulk CSF flow and/or egress. Further studies are required to understand Aβ accumulation in and around the ChP, and the role the blood–CSF barrier plays in global Aβ clearance. Together, the present findings suggest that CSF egress, and not production, may be related to Aβ plaque formation.

The present findings extend our previous work showing age-related changes to PSD volume, ChP perfusion and CSF flow by applying these methods in an ageing human population with varying levels of Aβ proteinopathy. Previous studies of CSF clearance in humans have utilized invasive methods such as intrathecal injection of MRI contrast agents or intravenous injection of PET radiotracers.^16,17,42,43^ Our novel techniques offer a non-contrasted, non-invasive method to capture indirect measures at both sites of production and egress of the CSF clearance pathway that can be readily implemented at other sites. Additionally, we utilize deep-learning algorithms to accurately segment both the ChP and PSD with commonly used anatomical MR sequences that can be applied to various other disease populations. These methods may further our understanding of the glymphatic system by providing accessible tools to characterize these structures in larger populations.

The findings of the present study contribute further support for a role of CSF-mediated Aβ clearance, but further research is required to fully characterize this clearance pathway. For example, longitudinal studies of preclinical/prodromal patients are required to understand the chronology of changes in the PSD with respect to Aβ aggregation and cognitive decline. Additionally, our results suggest specific regional changes to the PSD that relate to Aβ accumulation; and understanding the topography of glymphatic egress may clarify the underpinnings of commonly seen hotspots of Aβ aggregation in the brain parenchyma and vasculature. This CSF-mediated clearance pathway is also not limited to Aβ clearance; and applying these non-invasive methods to other patient populations such as Parkinson’s disease, multiple systems atrophy and Huntington’s disease may further our understanding of waste clearance in neurodegenerative diseases marked by aggregated proteins.

Several limitations should be considered. While well characterized by cognitive assessment, PET and multi-contrast MRI, the present study sample size of 23 participants is modest and as such the findings will require additional, larger cohorts to understand the relevance of PSD hypertrophy of Aβ retention more fully. It is also noted that the diurnal activity of neurofluids varies throughout the day and is shown to be more active during sleep than in an awake state.^50^ We accounted for these fluctuations by ensuring that scans were between the times of 7:00 am and 9:00 am. Lastly, while ^11^C-PIB displays high diagnostic accuracy for the detection of Alzheimer’s disease, it is often marred by overestimation of Aβ levels using the late-uptake SUVr method. This becomes an even more important issue when attempting to assess relationships of Aβ levels with other quantitative measures. Therefore, we elected to use a dynamic PET imaging assessment and implemented the Wu SRTM2 to quantitatively assess Aβ levels.^38^

## Conclusion

We assessed surrogate measures of CSF production, flow and egress in a cohort of older adults with varying levels of Aβ by applying novel non-invasive MRI methods and analyses. Our findings suggest that hypertrophy of the parasagittal space may more closely relate to cerebral Aβ retention than ChP perfusion or bulk CSF net flow in older adults with Alzheimer’s disease.

## Supplementary Material

fcad128_Supplementary_Data

## Data Availability

Data will be made available upon reasonable request.

## Abbreviations

Aβ =: amyloid-β
BP_ND_ =: non-displaceable binding potential
CDR =: Clinical Dementia Rating
ChP =: choroid plexus
F-CNN =: fully convolutional neural network
GM =: grey matter
ISF =: interstitial fluid
MoCA =: Montreal Cognitive Assessment
PIB =: Pittsburgh Compound B
PSD =: parasagittal dural
RBANS =: Repeatable Battery for the Assessment of Neuropsychological Status
SRTM2 =: simplified reference tissue model 2
SUVr =: standardized uptake value ratio
WM =: white matter
