# Supplementary material for: Parasagittal dural space hypertrophy and amyloid-β deposition in Alzheimer’s disease
Source: Brain Commun. 2023 Apr 18;5(3):fcad128. doi: 10.1093/braincomms/fcad128 (PMC10152899; doi:10.1093/braincomms/fcad128)
Supplement: fcad128_Supplementary_Data [file fcad128_supplementary_data.docx]

**Supplementary Table 1.** A table containing the AssemblyNet structures, and the general regions the structures belong to, used to create the mask for global Aβ burden quantification.

| **Region** | **AssemblyNet Structure** |
| --- | --- |
| Frontal | Anterior orbital gyrus |
|  | Frontal pole |
|  | Gyrus rectus |
|  | Lateral orbital gyrus |
|  | Medial orbital gyrus |
|  | Middle frontal cortex |
|  | Middle frontal gyrus |
|  | Opercular inferior frontal gyrus |
|  | Orbital inferior frontal gyrus |
|  | Posterior orbital gyrus |
|  | Subcallosal area |
|  | Superior frontal gyrus |
|  | Superior frontal gyrus medial |
|  | Triangular inferior frontal gyrus |
| Parietal | Angular gyrus |
|  | Postcentral gyrus |
|  | Postcentral gyrus medial segment |
|  | Precuneus |
|  | Superior parietal lobule |
|  | Supramarginal gyrus |

| **Region** | **AssemblyNet Structure** |
| --- | --- |
| Temporal | Entorhinal area |
|  | Fusiform gyrus |
|  | Inferior temporal gyrus |
|  | Middle temporal gyrus |
|  | Parahippocampal gyrus |
|  | Planum polare |
|  | Planum temporale |
|  | Superior temporal gyrus |
|  | Temporal pole |
|  | Transverse temporal gyrus |
| Insula | Anterior insula |
|  | Posterior insula |
| Dorsal striatum | Caudate |
|  | Putamen |

**Supplementary Table 2.** Results from Spearman’s and partial correlation analyses with global Aβ burden.

|  |  | | | Partial Correlation | | | | |
| --- | --- | --- | --- | --- | --- | --- | --- | --- |
|  |  | Spearman’s Correlation | |  |  | 95% Confidence Interval | |  |
| Variable | N | r | *p*-value | r | *p*-value | Lower Bound | Upper Bound | df |
| ChP Perfusion (mL/100g/min) | 22 | 0.079 | 0.748 | 0.272 | 0.245 | -0.19 | 0.64 | 18 |
| Net CSF Flow (mL/min) | 20 | -0.105 | 0.668 | 0.121 | 0.631 | -0.37 | 0.56 | 16 |
| PSD Volume - Total (cm^3^) | 23 | **0.529*** | **0.010** | **0.514*** | **0.017** | 0.11 | 0.75 | 19 |
| PSD Volume - Prefontal (cm^3^) | 23 | 0.279 | 0.197 | 0.257 | 0.260 | -0.20 | 0.62 | 19 |
| PSD Volume - Frontal (cm^3^) | 23 | **0.527*** | **0.010** | **0.562**** | **0.008** | 0.17 | 0.80 | 19 |
| PSD Volume - Parietal (cm^3^) | 23 | **0.616**** | **0.002** | **0.523*** | **0.015** | 0.12 | 0.78 | 19 |
| PSD Volume - Occipital (cm^3^) | 23 | 0.099 | 0.653 | -0.158 | 0.493 | -0.55 | 0.29 | 19 |

* = *p*-value < 0.05; ** = *p*-value < 0.01; df = degrees of freedom; ChP = choroid plexus; CSF = cerebrospinal fluid; PSD = parasagittal dural space

a. Partial correlations were covaried for age and sex.
